# Supplementary material for: Computational analysis of the effect of a binding protein (RbpA) on the dynamics of Mycobacterium tuberculosis RNA polymerase assembly
Source: PLoS One. 2025 Jan 30;20(1):e0317187. doi: 10.1371/journal.pone.0317187 (PMC11781615; doi:10.1371/journal.pone.0317187)
Supplement: S1 Text — This file also contains Graphical Abstract, S1 Table and S1–S12 Figs. (PDF) [file pone.0317187.s001.pdf]

1 | SUPPLEMENTARY INFORMATION

1.1 | Graphical Abstract

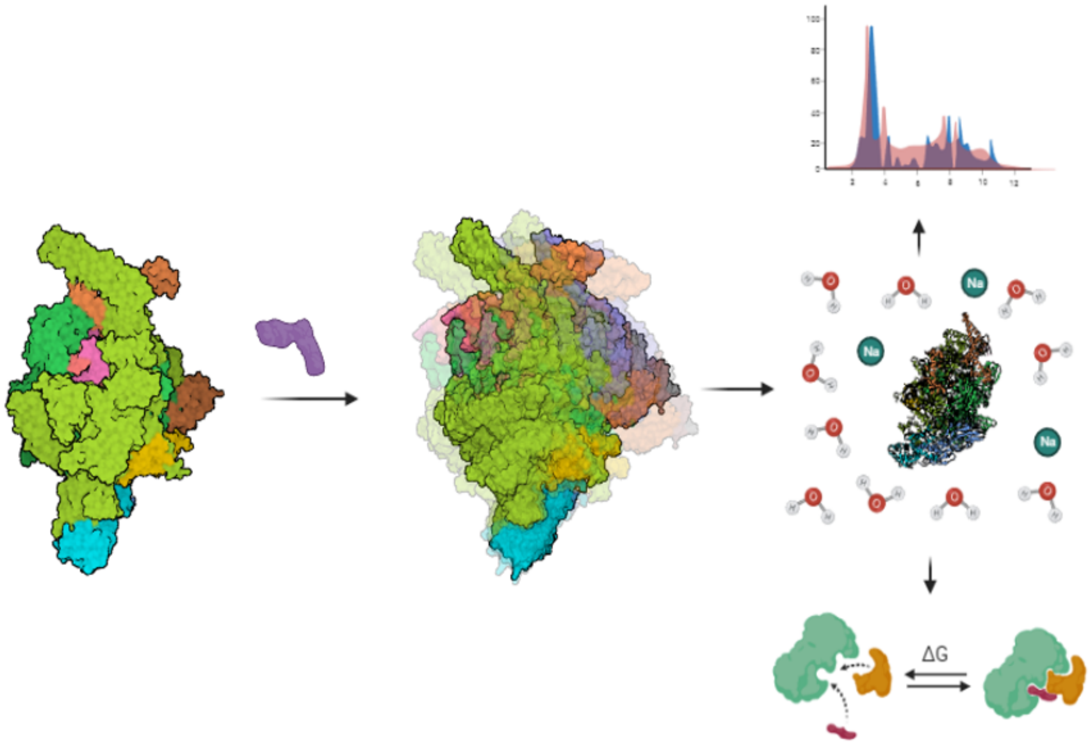

The computational investigation reveals the structural and dynamic changes induced by the binding of RbpA on RNA polymerase assembly. Using molecular dynamics simulations and other computational methods, the study highlights how RbpA modulates the conformational flexibility and communication pathways within RNAP. Key interactions between RbpA and the RNAP subunits are identified, showcasing their impact on enhancing the assembly stability and functional dynamics of the transcription machinery. The results provide molecular insights into the regulatory role of RbpA in RNAP activity.

1.2 | Transcription Process and the Role of RbpA in M. tuberculosis

In transcription, the genetic information encoded in a DNA strand is transcribed into a complementary messenger RNA (mRNA) molecule. This process occurs in three major stages: initiation, elongation, and termination. Bacteria rely on transcription and translation to rapidly respond to environmental changes. In contrast to eukaryotes, which utilize three distinct RNA polymerases, bacteria, including *Mycobacterium tuberculosis (M. tb)*, possess a single RNA polymerase (RNAP) to carry out transcription.

In M. tb, RNAP catalyzes RNA synthesis and is composed of five subunits: α1, α2, β, β', and ω (Figure S1). The association of the sigma factor (σ) with the core enzyme transforms the RNAP apoenzyme into the holoenzyme, enabling promoter recognition and transcription initiation. RbpA, a transcriptional activator, binds tightly to the housekeeping σ factors in M. tb[1]. RbpA is a small protein (13 kDa) composed of several domains, including the N-terminal tail (NTT), central core domain (CD), σ-interacting domain (SID), and a flexible basic linker (BL) loop[1,2].

Transcription initiation begins when the RNAP holoenzyme (R) binds to a promoter sequence (P), forming the closed promoter complex (RPc). The binding is driven by free energy changes that facilitate multiple conformational rearrangements in both RNAP and the promoter DNA, leading to the transition from the closed (RPc) to the open promoter complex (RPo). Once in the open complex, RNAP initiates RNA polymerization by incorporating nucleotides complementary to the template DNA strand, forming the growing RNA transcript.

RbpA binds to the β subunit of RNA polymerase (RNAP) and forms a stable complex with the N-terminal domain of σB, facilitated through both its N- and C-terminal regions. Specifically, the RbpA's SID region interacts with the σA2 domain, while its BL domain makes contact with the phosphate backbone of promoter DNA, just upstream of the -10 element. Additionally, the C-terminal domain of RbpA engages with the Zinc-Binding Domain of the RNAP β' subunit. Notably, conserved residues in the RbpA N-terminal tail (RbpANTT) interact with conserved residues in the σ-finger (σ3.2-linker) along one side of the channel and also make contacts with conserved residues in both the ZBD and the β'lid on the opposite side.

CarD, on the other hand, primarily associates with the β1-lobe of the RNAP β subunit, positioning itself near the upstream region of the transcription bubble during the formation of the RNA polymerase-promoter open complex (RPo). Structural analyses reveal that CarD binds to the unique junction of double-stranded/single-stranded (ds/ss) DNA within the upstream portion of the transcription bubble. Although CarD does not make significant contacts with σA, it interacts with promoter DNA from positions -14 to -10 via the minor groove on the distorted side of the DNA. Despite these interactions, the overall structure of the transcription bubble and the σA/DNA interactions remain unchanged between RPo and CarD/RPo complexes. Furthermore, direct contacts between the N-terminal ends of CarD-CTD α-helices and the promoter DNA help stabilize these interactions. These binding patterns suggest that both RbpA and CarD play essential roles in modulating transcription initiation through their distinct binding sites on RNAP.

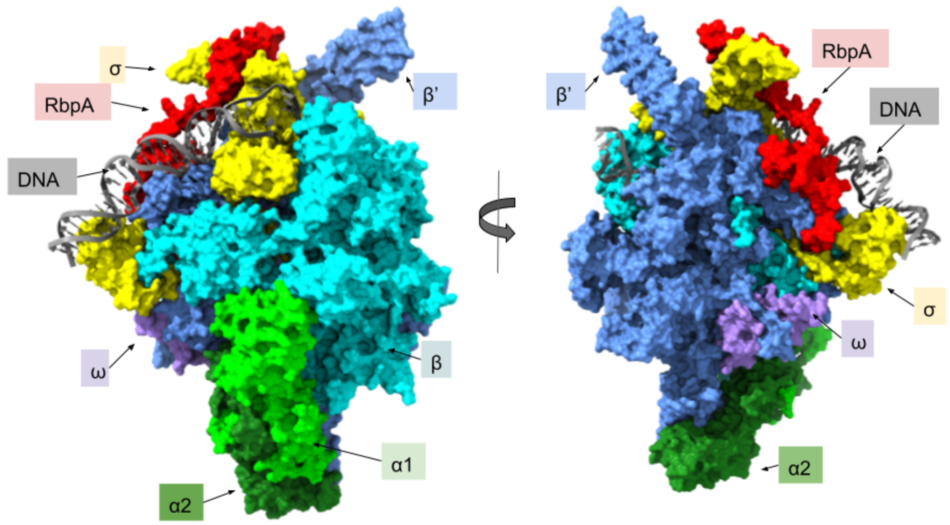

Fig S1 RNA polymerase complex (M.tb) and its subunits α1, α2, β, β', and ω along with σ, RbpA and DNA

1.3 | Materials and methods

1.3.1 | Dataset preparation

During dataset preparation, we identified 20 available structures of RNA polymerase. Of these, only 4 contained RbpA, and 4 included both RbpA and CarD. We selected structures from this subset based on the highest resolution and the fewest missing residues. From the remaining 12 structures, only 5 were unbound to ligands (such as fidaxomicin and rifampicin). From these, we further selected a structure using similar criteria of resolution and missing residues. Details of the structures are provided in 1 For other details on methodology please refer to[3].

1.3.2 | Molecular dynamics simulations

The prepared systems were solvated in cubic boxes, with a 10 Å cutoff from each edge, to minimize interactions with periodic images. Na+ and Cl- ions were added to neutralize the systems. System preparation was performed using the CHARMM-GUI server[4,5] and the CHARMM c36m force field. Explicit water was modeled using TIP3P[6] parameters. The systems were then

Table 1 S1: Dataset

PDB ID	5UH5	6C04	6EDT
**Description**	RbpA unbound structure	RbpA bound structure	RbpA and CarD bound structure
**Experiemental technique**	X-ray crystallography	Cryo-EM	Cryo-EM
**Resolution**	3.75Å	3.27Å	3.9Å
**Year of publication**	2017	2018	2019
**DNA**	Fragments	Fragments	Open promoter

minimized for 5000 steps with a tolerance of 100 kJ/mol. Equilibration was carried out for 125 ps at a temperature of 303 K. Subsequently, three replicates of MD simulations were performed for each system, resulting in a total simulation time of 300 ns. Periodic boundary conditions were applied, and long-range electrostatic interactions were computed using the particle mesh Ewald algorithm. Lennard-Jones interactions were employed with cutoff distances of 1.0 to 1.2 nm, based on the CHARMM force field. Bonds involving hydrogen atoms were constrained using the SHAKE algorithm. Simulations were conducted using a Langevin thermostat at 303.15 K. The time step for equilibration was set to 1 fs, while for the production runs, it was 2 fs, and trajectory snapshots were saved every 10 ps.

The root-mean-square deviation (RMSD) and root-mean-square fluctuation (RMSF) were calculated to assess the overall structural stability and flexibility of the proteins. The Cα RMSD values were computed after aligning the Cα atoms of the proteins. RMSF values were determined for the Cα atoms to quantify the local residue fluctuations. Angles and contact analysis were also performed using MDAnalysis. Contacts between protein pairs and DNA were evaluated by averaging the distances over the trajectories for each residue pair within a 5 Å threshold. Energy calculations were conducted using the molecular mechanics generalized Born and surface area (MMPB/SA) approach[7] with the gmx_MMPBSA package[8]. Total binding energies, as well as electrostatic and van der Waals (vdW) contributions, were computed for protein-DNA pairs, excluding the entropic contribution. By employing this comprehensive MD simulation and analysis protocol, we aimed to gain insights into the dynamic behavior, structural stability, fluctuations, intermolecular contacts, and binding energies within the RNA polymerase complexes and their interactions with auxiliary factors.

1.3.3 | Perturbation response scanning

Allosteric regulation of protein function is critical in regulating cellular processes[9,10,11] so its underlying mechanisms are of primary concern for protein engineering[12] and drug design[13]. Allostery can happen in many ways; generally, it involves an effector modifying the protein's behaviour at the active site. Effectors can either enhance or decrease the activity of a protein. The general mechanism of allostery is based predominantly on mechanical operations at the molecular and atomic scales. The long-range nature of allosteric communication is often well-described by low-frequency modes that involve the motion of the majority of atoms[14]. The response of effectors was measured through the determination of kinetic parameters. This can be achieved by applying linear response theory (LRT) to proteins under selected external perturbations[15].

1.3.4 | Virtual screening of inhibitors

For the docking studies, we utilized two compound libraries: FDA-approved drugs and the Super Natural II database, which contains natural products. Natural products are known to play a significant role in drug discovery, as many share topological pharmacophore patterns with commercially available drugs. Understanding the physicochemical and structural features of these compounds is critical for drug development, which is why the Super Natural database was selected for this study. We chose FDA-approved drugs because their safety profiles and pharmacokinetic properties have already been well-characterized, making them valuable candidates for repurposing in computational approaches to inhibitor design. By focusing on compounds that are already approved, we aim to streamline the process of identifying potential inhibitors for RNA polymerase, increasing the likelihood of identifying viable drug candidates for further development.

1.4 | Results

1.5 | Significant structural changes at the interface were observed in RNA polymerase upon interaction with RbpA

Among all the subunits, β, β' and σ show major Cα deviations, and these range from 0-10Å. Intriguingly, we also observed structural changes at sites distant from the interface. For instance, in subunitα1, the Cα deviation is greater than 3Å in regions 150-160 and 180-190 which are far from the interface of RbpA. Since these differ from the trends observed on global-RMSD, specific interface regions were analyzed further.

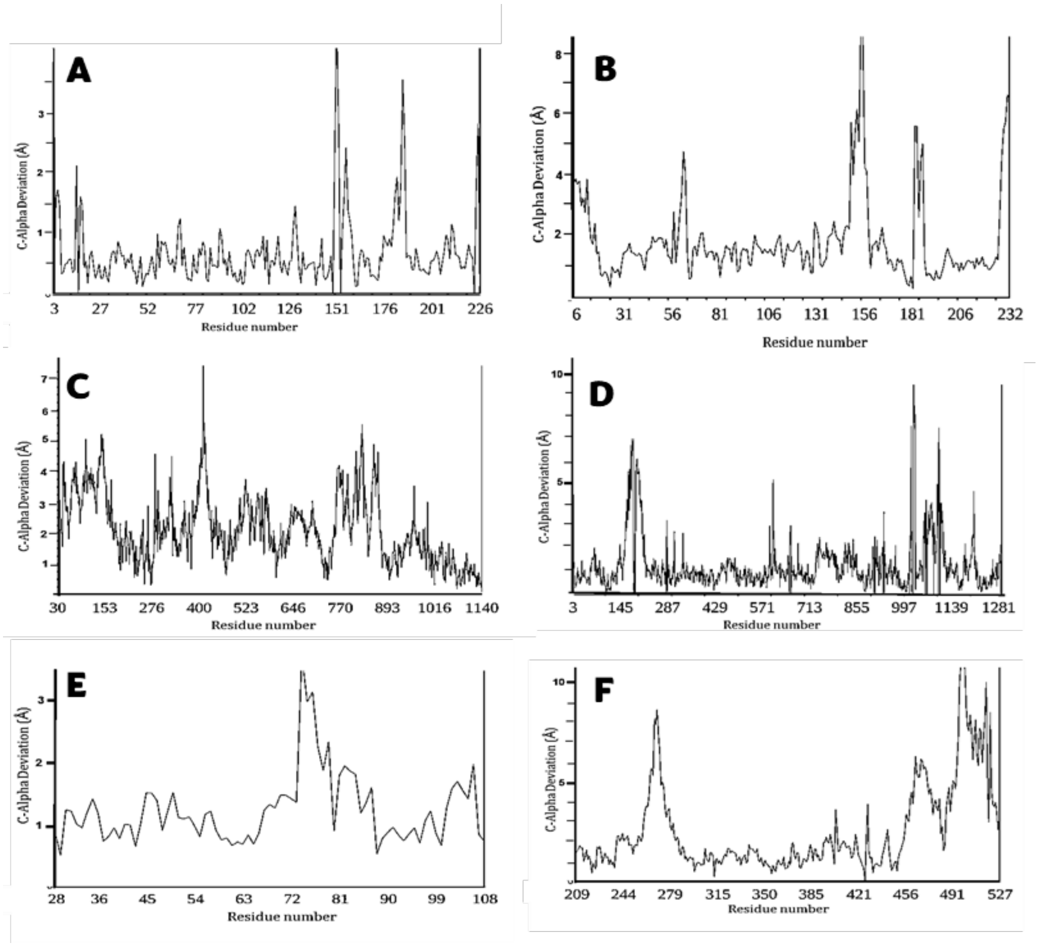

Fig S2 Cα deviation for all the subunits in RNA polymerase complex. A) α1, B) α2, C) β, D) β', E) ω and F) σ-factor

To gain insight into the binding mechanism of RbpA, we examined the occurrence of various interactions (Electrostatic, Hydrophobic, and salt bridges) among all subunits (Figure S3 B, C, and D), which exhibited consistent trends with the interfacial residue analysis. Moreover, the analysis of the interaction area provided further insights into the mode of interaction. Some subunits demonstrated an increase in the interaction area, while others displayed a decrease.

Notably, significant alterations were observed in the electrostatic and hydrogen bond energies, suggesting their involvement in DNA interactions.

1.6 | RbpA binding does not affect large domain motions

Normal mode analysis helps in understanding the dynamic behaviour of proteins deduced using physical laws of motion. Figure S5 shows square fluctuations obtained using the NMA. From the plots, it can be observed that there are regions in every subunit

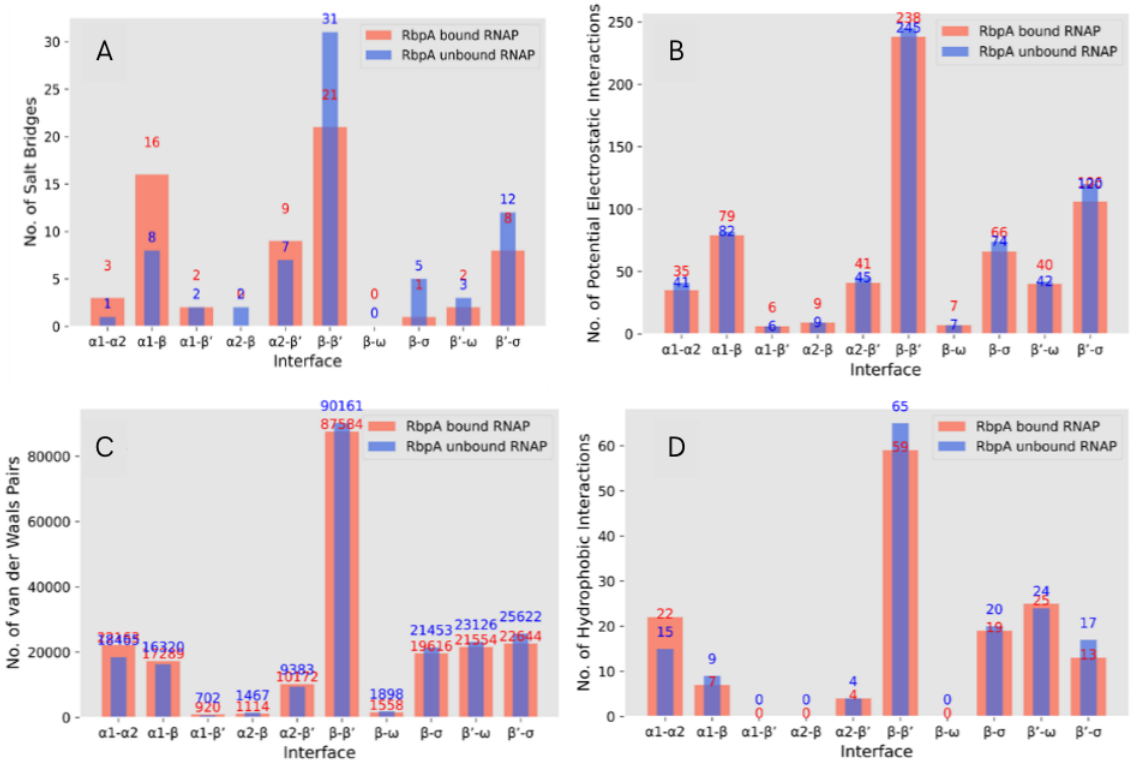

Fig S3 Analysis of interfacial properties among various subunits. X-axis is the name of the interface and on the Y-axis there is the number of interactions or Area of Interaction (Å). A) Number of salt bridges, B)Number of potential electrostatic interactions C) Number of Van der Waals pairs, and D) Number of hydrophobic interactions

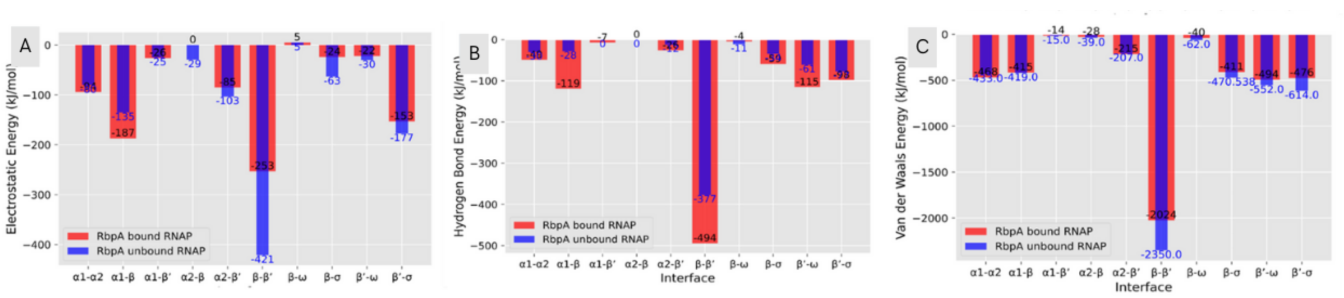

Fig S4 Analysis of interaction energy among various subunits. X-axis contains the names of the interfaces while Y-axis has the energies (Kj/mol). A) Total stabilization energy, B) Electrostatic energy, C) Hydrogen bond energy and D) Van der Waals energy.

with higher dynamics than other regions in the proteins. All these regions are the interface regions with other proteins in the complex. A detailed analysis of these regions is discussed in the below sections. It can also be observed that there are regions in the proteins β, β', and σ that show higher fluctuations upon interaction with RbpA. From our analysis, we could identify changes in the fluctuations even in the proteins that are not involved directly in RbpA interaction. I could not find any differences in terms of the cross-correlation matrix. It can be inferred that RbpA does not change correlations of residue motions of RNA polymerase.

The binding of DNA improves the stability of the RNA polymerase complex. Using Molecular Dynamics simulations, we were able to study the square fluctuations of all subunits in all the RNA polymerase complexes. The RMSD plot depicting the same was shown in Figure S6. A notable trend was observed, indicating that the absence of DNA within the complexes imparts a higher degree of dynamic behavior. Specifically, the RMSD values obtained for the DNA-bound complexes were comparatively

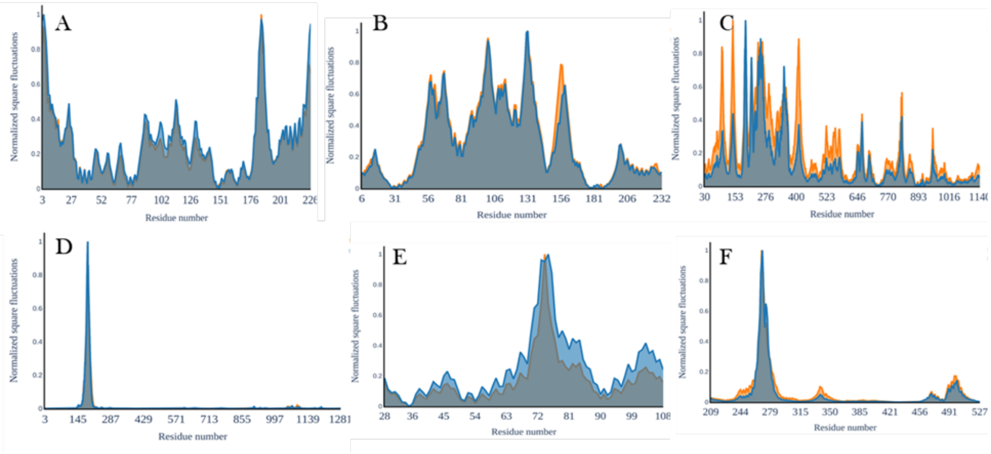

Fig S5 Normalized square fluctuations were obtained from Normal mode analysis. X-axis represents the residue number and Y-axis shows the normalized square fluctuations. A) $\alpha 1$, B) $\alpha 2$, C) β, D) β', E) ω, and F) σ.

lower than those observed in the complexes lacking DNA. This intriguing observation underscores the crucial role played by DNA binding in conferring stability upon the RNAP complex.

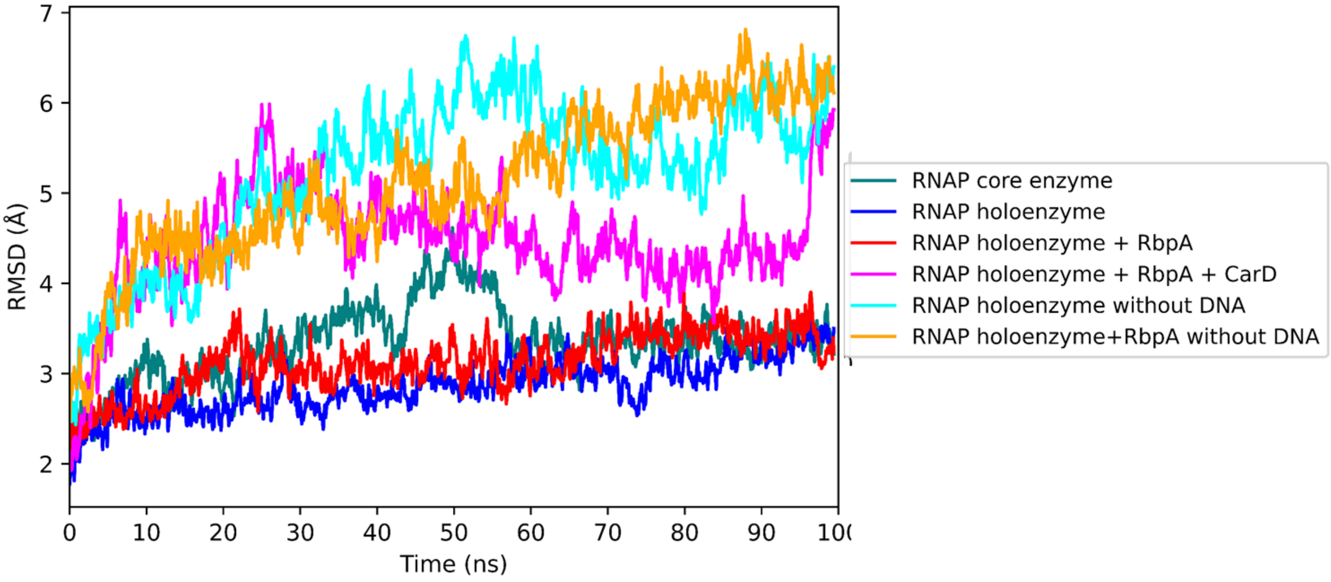

Fig S6 RMSD plot obtained from molecular dynamics analysis. X-axis represents the time in ns and Y-axis shows the RMSD in Å. Legend shows colour-coded names of complexes.

1.7 | Dynamic regulation of RNA Polymerase β and β' subunits by RbpA and CarD

β subunit is a 150kDa protein, which has a double-psi beta-barrel motif in the active site. The β subunit interacts with Mg (II) ions and facilitates the nucleotidyl transfer reaction [16]. The $\beta 1$ domain (47-172 and 375-428) and $\beta 2$ domain (177-370) dynamics are assumed to be tightly regulated by CarD and RbpA, as they form the pivot for RNAP activity [17]. CarD interacts with both the $\beta 1$ and $\beta 2$ domains, while RbpA binds to residues 478-677 of the β subunit. Furthermore, the β flap, which is essential for promoter recognition, encompasses residues 855-914 and interacts with the σ domain.

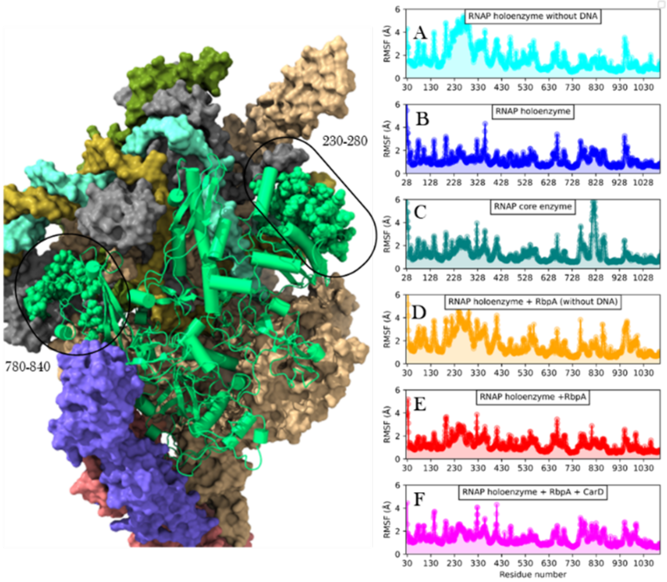

Fig S7 RMSF plots of β obtained from molecular dynamics analysis. X-axis represents the residue number and Y-axis shows the RMSF in Å. A) RNAP holoenzyme without DNA, B) RNAP holoenzyme, C) RNAP core enzyme, D) RNAP holoenzyme with RbpA but without DNA, E) RNAP holoenzyme with RbpA and DNA, and F) RNAP holoenzyme with RbpA, CarD, and DNA. On the left is the visual representation of the residues, showing significant differences in the fluctuations. β is shown in the cartoon with highlighted residues as spheres.

Our observations reveal important insights into the dynamics of the β subunit (Figure S7). In the absence of the σ factor, we found that the β-subunit flap exhibits high flexibility, which may pose challenges for efficient promoter recognition. Additionally, a region of the β subunit (residues 230-280), which interacts with the σ factor, displays high flexibility in the absence of DNA, suggesting its role in DNA interactions. Importantly, both RbpA and CarD are shown to regulate the dynamics of the β subunit, promoting its stability and facilitating interactions with DNA. Specifically, CarD, in conjunction with RbpA, enhances fluctuations in a specific region of the $\beta 1$ domain, whereas RbpA alone does not induce significant changes in this region. These findings highlight the critical role of RbpA and CarD in modulating the dynamics of the β subunit, which in turn affects the stability of the RNA polymerase complex and its interactions with DNA. By regulating the dynamics of the β subunit, RbpA and CarD contribute to the overall efficiency and accuracy of the transcription process.

1.8 | RbpA Impacts RNA Polymerase ω Subunit Dynamics

The ω subunit of RNA polymerase (RNAP) has received relatively less attention in terms of detailed studies compared to other subunits. However, there has been limited literature on this: for example, the presence of the ω subunit in RNAP contributes to its ability to rapidly recover from denaturing conditions [18] suggesting its potential role in maintaining the structural integrity and functional activity of the enzyme. Secondly, studies involving the elimination of theω subunit in mycobacterial RNAPs have revealed significant disruptions in protein assembly and compromised RNAP activity [19]. These observations suggest that the ω subunit in mycobacteria may possess essential functions that distinguish it from the ω subunit found in other organisms.

Our analysis of the ω subunit of RNA polymerase reveals that it exhibits a limited amount of flexibility, with only two distinct regions displaying notable dynamics, as revealed by current molecular dynamics studies (Figure S8). Specifically, the region spanning residues 70-78 demonstrates high flexibility and is involved in interactions with the β' subunit. Interestingly, this region remains highly flexible, regardless of the presence or absence of other factors. In contrast, the region spanning residues 83-90 exhibits flexibility primarily in the absence of RbpA, suggesting a potential regulatory role of RbpA in modulating the dynamics of this region.

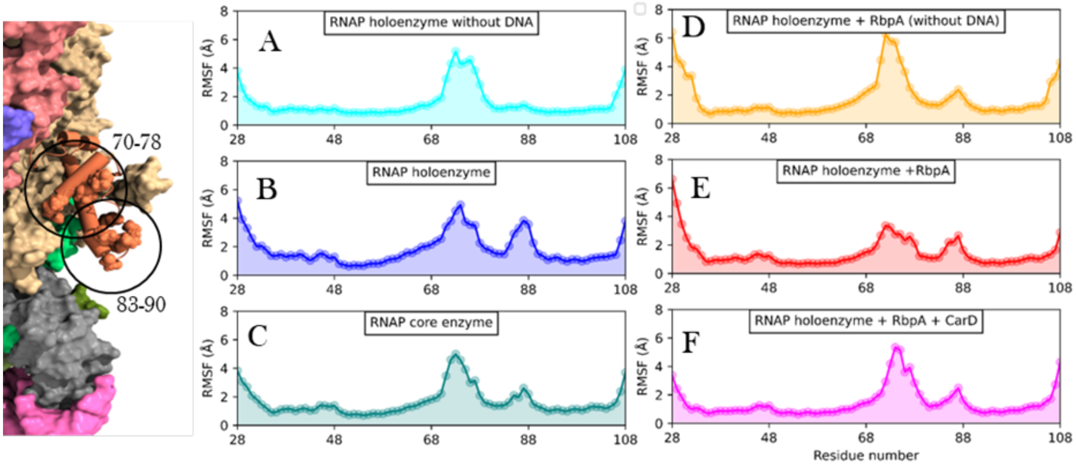

Fig S8 RMSF plots of ω obtained from molecular dynamics analysis. X-axis represents the residue number and Y-axis shows the RMSF in Å. A) RNAP holoenzyme without DNA, B) RNAP holoenzyme, C) RNAP core enzyme, D) RNAP holoenzyme with RbpA but without DNA, E) RNAP holoenzyme with RbpA and DNA, and F) RNAP holoenzyme with RbpA, CarD, and DNA. On the left is the visual representation of the residues showing significant differences in the fluctuations. ω is shown in the cartoon with highlighted residues as spheres. obtained from molecular dynamics analysis. X-axis represents the residue number and Y-axis shows the RMSF in Å. A) RNAP holoenzyme without DNA, B) RNAP holoenzyme, C) RNAP core enzyme, D) RNAP holoenzyme with RbpA but without DNA, E) RNAP holoenzyme with RbpA and DNA, and F) RNAP holoenzyme with RbpA, CarD, and DNA. On the left is the visual representation of the residues showing significant differences in the fluctuations. ω is shown in the cartoon with highlighted residues as spheres.

1.9 | Dynamics of σ subunit

The σ factor is known to bind reversibly to the RNA polymerase (RNAP) core and is essential for transcription initiation. Our analysis reveals that the presence of RbpA influences the dynamics of specific regions within the σ factor (Figure S10). Notably, RbpA decreases the flexibility of residues 260-280 within the σ factor, which are directly involved in interactions with RNAP core subunits. Conversely, RbpA enhances the flexibility of residues 480-510, which participate in interactions with both RbpA and DNA. These findings suggest a regulatory role of RbpA in modulating the dynamics of σ factor residues critical for its functional interactions with RNAP core and DNA, facilitating enhanced DNA binding, during transcription initiation.

1.10 | virtual screening

Top compound that interacts with site2 is the compound with MolProt id 039-338-204. It shows a promiscuous and sustained interaction with α subunit of RNA polymerase. The interactions are mainly hydrogen bonding, and the major contributors are residues Arg 182 and Arg 186 (Figure S11). There are other MolProt molecules that displayed medium interactions are 008-346-738, 002-515-942 and 028-610-204.

Top compound that interacts with site2 from FDA approved molecules is Icotinib. It is a potent epidermal growth factor receptor tyrosine kinase inhibitor. It is highly selective and binds to the ATP binding site of the EGFR protein. It interacts with α subunit mainly via h-bonding and has several residues contributing to these interactions (Figure S12). It also has a few residues (which include 62, 151 and 165) that are constantly interacting with the molecule throughout 100ns. Apart from these other FDA-molecules displayed medium interactions after simulation studies are namely cabozantnib, ceftolozane and irinotecan.

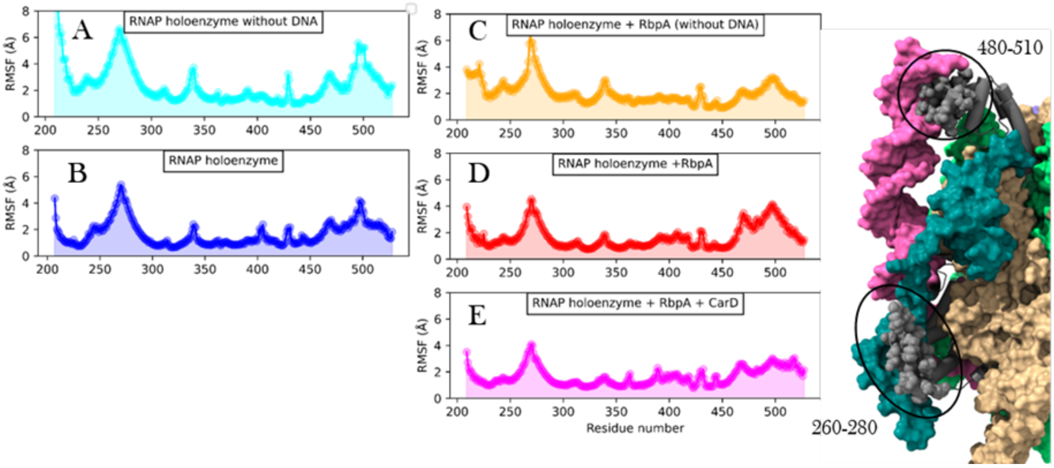

Fig S9 RMSF plots of σ obtained from molecular dynamics analysis. X-axis represents the residue number and Y-axis shows the RMSF in Å. A) RNAP holoenzyme without DNA, B) RNAP holoenzyme, C) RNAP core enzyme, D) RNAP holoenzyme with RbpA but without DNA, E) RNAP holoenzyme with RbpA and DNA, and F) RNAP holoenzyme with RbpA, CarD, and DNA. On the right is the visual representation of the residues showing significant differences in the fluctuations. σ is shown in the cartoon with highlighted residues as spheres.

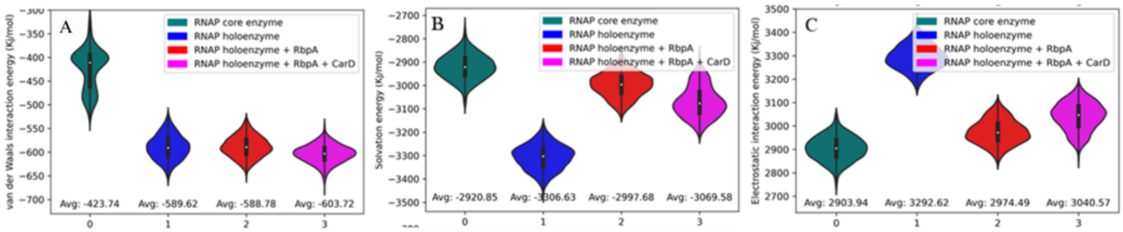

Fig S10 Analysis of interaction energy between complex and DNA obtained from molecular dynamics analysis. X-axis represents the complexes and Y-axis shows the energy in Kj/mol. A) Van der Waals energy, B) Solvation energy C) Electrostatic energy) .

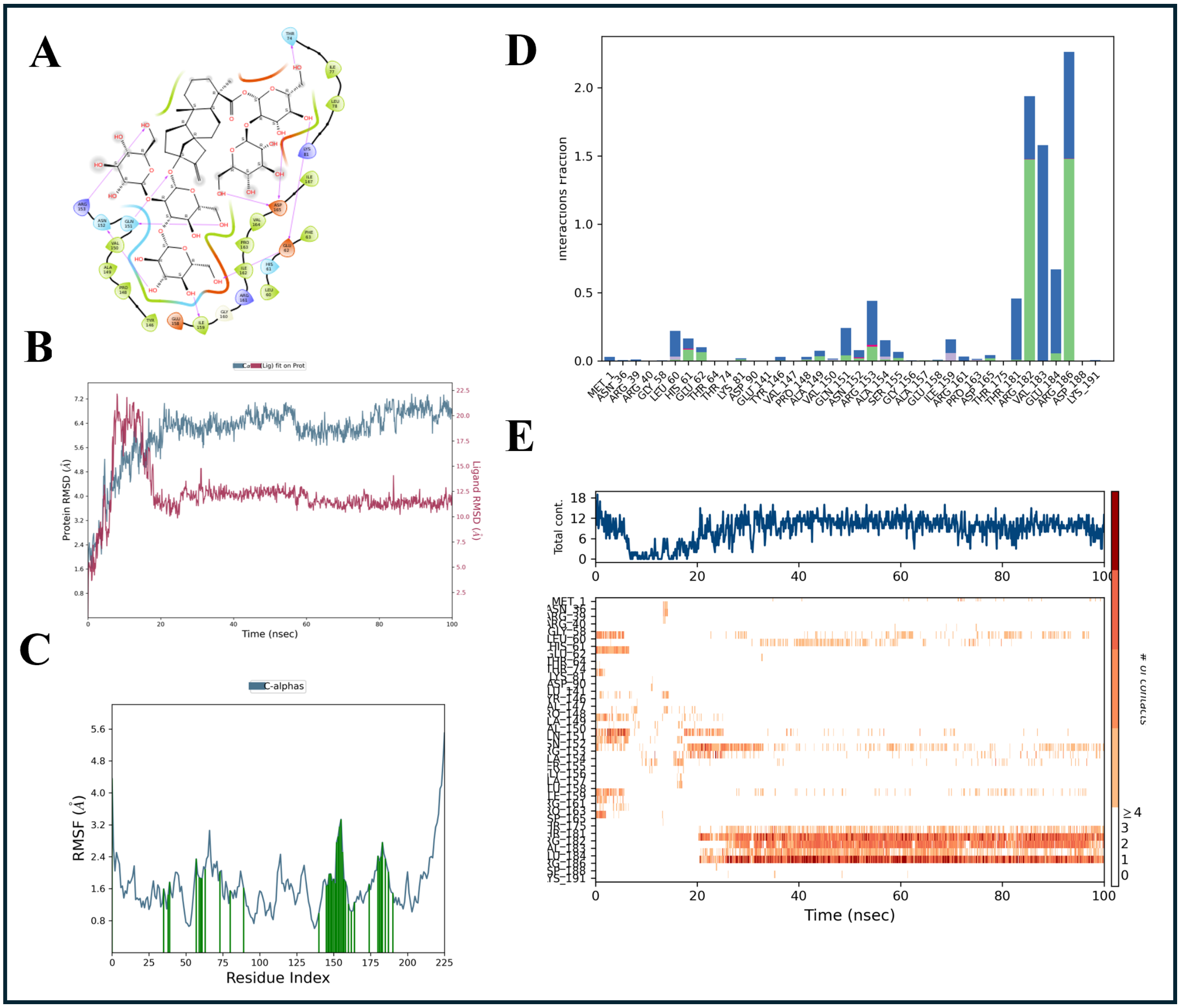

Fig S11 Analysis of the MD Simulation results of the MolProt compound with ID 039-338-204 with RNA polymerase. A) Pictoral representation of the compound bound to RNA Polymerase, B) RMSD plot of the protein and ligand during the simulation. X-axis is time and Y-axis represents the RMSD. C) RMSF plot of the α subunit during the simulation. X-axis is time and Y-axis represents the RMSF. D) Fraction of the interactions of a given residue with ligand during the simulations. E) Plot representing the contacts of residues of α subunit with ligand during the simulation.

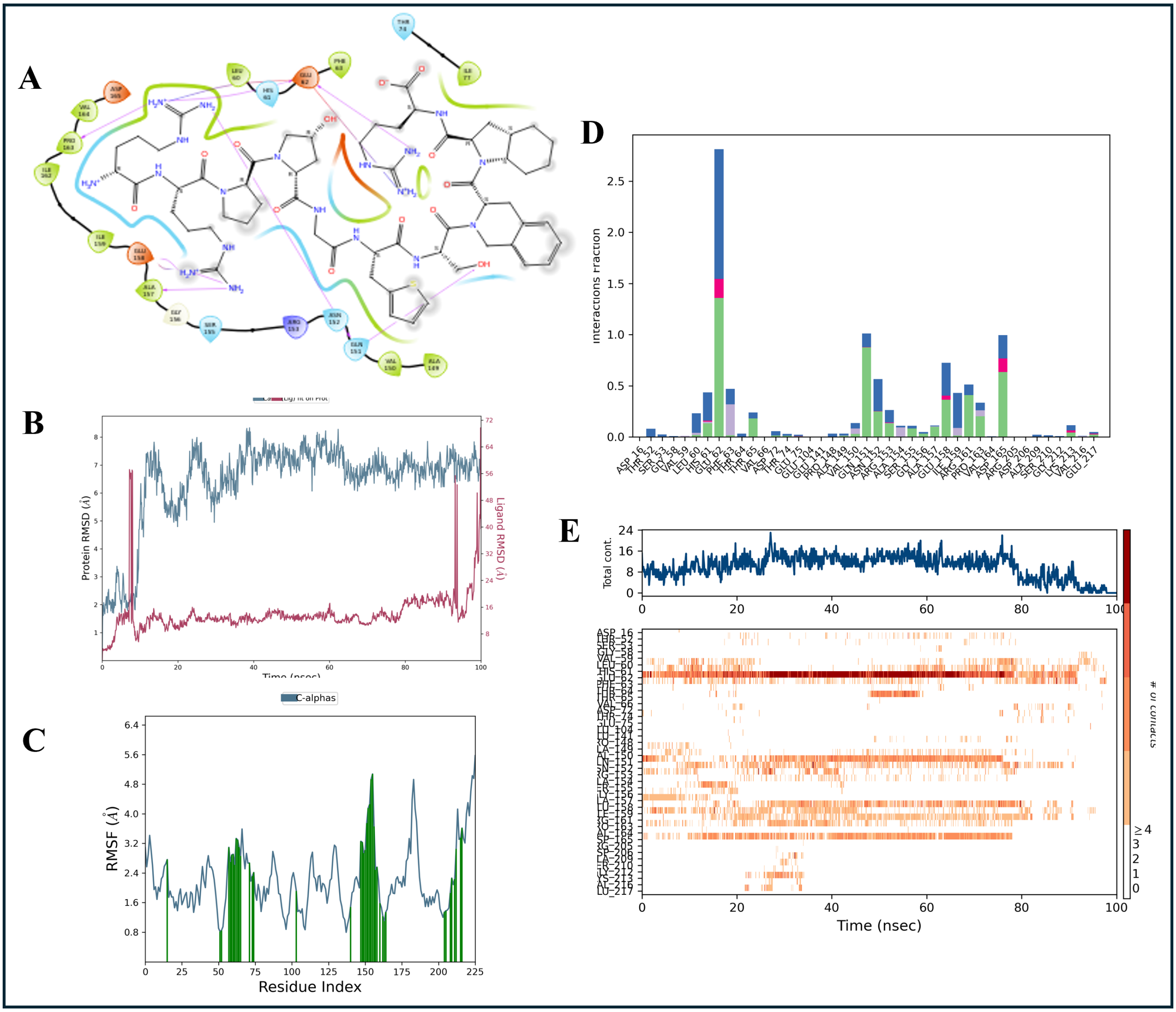

Fig S12 Analysis of the MD Simulation results of the Icotinib with RNA polymerase. A) Pictoral representation of the compound bound to RNA Polymerase, B) RMSD plot of the protein and ligand during the simulation. X-axis is time and Y-axis represents the RMSD. C) RMSF plot of the α subunit during the simulation. X-axis is time and Y-axis represents the RMSF. D) Fraction of the interactions of a given residue with ligand during the simulations. E) Plot representing the contacts of residues of α subunit with ligand during the simulation.

References

1. Bortoluzzi A, Muskett FW, Waters LC, et al. Mycobacterium tuberculosis RNA polymerase-binding protein A (RbpA) and its interactions with sigma factors. *Journal of Biological Chemistry* 2013; 288(20): 14438–14450. doi: 10.1074/JBC.M113.459883

2. Hubin EA, Tabib-Salazar A, Humphrey LJ, et al. Structural, functional, and genetic analyses of the actinobacterial transcription factor RbpA. *Proceedings of the National Academy of Sciences of the United States of America* 2015; 112(23): 7171–7176. doi: 10.1073/PNAS.1504942112

3. Bheemireddy S, Srinivasan N. Computational Study on the Dynamics of Mycobacterium Tuberculosis RNA Polymerase Assembly. *Springer US* 2022: 61–79. doi: 10.1007/978-1-0716-2413-5_5

4. Jo S, Kim T, Iyer VG, Im W. CHARMM-GUI: A web-based graphical user interface for CHARMM. *Journal of Computational Chemistry* 2008; 29(11): 1859–1865. doi: 10.1002/JCC.20945

5. Jo S, Cheng X, Lee J, et al. CHARMM-GUI 10 years for biomolecular modeling and simulation. *Journal of Computational Chemistry* 2017; 38(15): 1114–1124. doi: 10.1002/JCC.24660

6. Price DJ, Brooks CL. A modified TIP3P water potential for simulation with Ewald summation. *The Journal of Chemical Physics* 2004; 121(20): 10096–10103. doi: 10.1063/1.1808117

7. Sheng YJ, Yin YW, Ma YQ, Ding HM. Improving the Performance of MM/PBSA in Protein-Protein Interactions via the Screening Electrostatic Energy. *Journal of Chemical Information and Modeling* 2021; 61(5): 2454–2462. doi: 10.1021/ACS.JCIM.1C00410/SUPPL_FILE/CI1C00410_SI_002.ZIP

8. Valdés-Tresanco MS, Valdés-Tresanco ME, Valiente PA, Moreno E. Gmx_MMPBSA: A New Tool to Perform End-State Free Energy Calculations with GROMACS. *Journal of Chemical Theory and Computation* 2021; 17(10): 6281–6291. doi: 10.1021/ACS.JCTC.1C00645/ASSET/IMAGES/LARGE/CT1C00645_0005.JPEG

9. Nussinov R. Introduction to Protein Ensembles and Allostery. *Chemical Reviews* 2016; 116(11): 6263–6266. doi: 10.1021/ACS.CHEMREV.6B00283/ASSET/IMAGES/LARGE/CR-2016-00283T_0001.JPEG

10. Nussinov R, Tsai CJ. Allostery in disease and in drug discovery. *Cell* 2013; 153(2): 293–305. doi: 10.1016/J.CELL.2013.03.034

11. Wodak SJ, Paci E, Dokholyan NV, et al. Allostery in Its Many Disguises: From Theory to Applications. 2019

12. Raman S, Taylor N, Genuth N, Fields S, Church GM. Engineering allostery. 2014

13. Gaczynska M, Osmulski PA. Harnessing Proteasome Dynamics and Allostery in Drug Design. *Antioxidants & Redox Signaling* 2014; 21(17): 2286–2301. doi: 10.1089/ars.2013.5816

14. Tzeng SR, Kalodimos CG. Protein dynamics and allostery: An NMR view. 2011

15. Ikeguchi M, Ueno J, Sato M, Kidera A. Protein structural change upon ligand binding: Linear response theory. *Physical Review Letters* 2005; 94(7): 078102. doi: 10.1103/PhysRevLett.94.078102

16. Sutherland C, Murakami KS. An Introduction to the Structure and Function of the Catalytic Core Enzyme of Escherichia coli RNA Polymerase . *EcoSal Plus* 2018; 8(1). doi: 10.1128/ECOSALPLUS.ESP-0004-2018

17. Stephanie F, Sumo U, Tambunan F, Siahaan TJ. M. tuberculosis Transcription Machinery: A Review on the Mycobacterial RNA Polymerase and Drug Discovery Efforts. *mdpi.com* 2022; 12(11). doi: 10.3390/life12111774

18. Patel U, Gautam S, Biomolecules DC, 2020 u. Validation of omega subunit of RNA polymerase as a functional entity. *mdpi.com* 2020; 10(11): 1–11. doi: 10.3390/biom10111588

19. Mao C, Zhu Y, Lu P, Feng L, Chen S, Hu Y. Association of ω with the Cterminal region of the β' subunit is essential for assembly of RNA polymerase in Mycobacterium tuberculosis. *Journal of Bacteriology* 2018; 200(12). doi: 10.1128/JB.00159-18
